# Spatial and Spectral Auditory Temporal-Order Judgment (TOJ) Tasks in Elderly People Are Performed Using Different Perceptual Strategies

**DOI:** 10.3389/fpsyg.2018.02557

**Published:** 2018-12-11

**Authors:** Elzbieta Szelag, Katarzyna Jablonska, Magdalena Piotrowska, Aneta Szymaszek, Hanna Bednarek

**Affiliations:** ^1^Laboratory of Neuropsychology, Nencki Institute of Experimental Biology of Polish Academy of Sciences, Warsaw, Poland; ^2^Faculty of Psychology, SWPS University of Social Sciences and Humanities, Warsaw, Poland

**Keywords:** temporal information processing, spatial task, spectral task, temporal-order judgment, aging, auditory temporal-order threshold

## Abstract

The Temporal-Order Judgment (TOJ) paradigm has been widely investigated in previous studies as an accurate measure of temporal resolution and sequencing abilities in the millisecond time range. Two auditory TOJ tasks are often used: (1) a spatial TOJ task, in which two identical stimuli are presented in rapid succession monaurally and the task is to indicate which ear received the first stimulus and which ear received the second one (*left-right* or *right-left*), and (2) a spectral TOJ task, in which two tones of different frequencies are presented asynchronously to both ears binaurally and the task is to report the sequence of these tones (*low-high* or *high-low*). The previous literature studies conducted on young volunteers indicated that the measured temporal acuity on these two tasks depended on the procedure used. As considerable data are now available about age-related decline in temporal resolution ability, the aim of the present study was to compare in elderly subjects the pattern of performance on these two tasks. A total of 40 normal healthy volunteers aged from 62 to 78 years performed two TOJ tasks. The measurement was repeated in two consecutive sessions. Temporal resolution was indexed by the Auditory Temporal-Order Threshold (ATOT), i.e., the minimum time gap between successive stimuli necessary for a participant to report a *before-after* relation with 75% correctness. The main finding of the present study was the indication of differences in the elderly in performance on two tasks. In the spatial task, the distribution of obtained ATOT values did not deviate from the Gaussian distribution. In contrast, the distribution of data in the spectral task deviated significantly from the Gaussian and was spread more to the right. Although lower ATOT values were usually observed in Session 2 than in Session 1, such difference was significant only in the spectral task. We conclude that although temporal acuity and sequencing abilities in the millisecond time range are probably based in neuronal oscillatory activity, the measured ATOTs in the elderly seem to be stimulus-dependent, procedure-related, and influenced by the perceptual strategies used by participants.

## Introduction

For over three decades, an increasing number of experimental studies have suggested that Temporal Information Processing (TIP) is an essential component of human cognition. Researchers have been interested in this topic because of converging evidence indicating that patterning in time plays a fundamental role in human behavior, as many mental functions display specific temporal dynamics (Pöppel, 1994, 1997, 2009; Szelag et al., 2004a; Wittmann, 2009, 2011). Thus, patterning in time provides a structure for cognition and a framework for our working brains, proving that the brain incorporates the time dimension into its computation. Findings about differences in TIP among various clinical subgroups emphasize the importance of timing-cognition relations, as they can be understood as reflecting fundamental differences in TIP associated with deficient cognition (see Teixeira et al., 2013 for an overview). It seems, therefore, that cognitive processes cannot be understood without taking their time frame into account.

Existing evidence indicates that TIP is not a monolithic process. One may distinguish several time ranges controlled by specific neural mechanisms employing discrete time sampling. This study focuses on the millisecond time domain, which provides a structure for motor and sensory processing, including speech processing (Pöppel, 1997, 2009; Wittmann, 1999, 2009, 2011; Szelag et al., 2004a, 2008, 2010, 2011, 2014; Szelag and Dacewicz, 2016). This time domain is related to the perception of succession and the temporal order of events – distinct stimuli must be separated by some tens of milliseconds in order for them to be identified as different events.

The Temporal-Order Judgment (TOJ) paradigm is one of several psychophysical paradigms used to measure the efficiency of temporal resolution in this time domain. It reflects the ability to perceive the temporal order of (at least two) stimuli presented in rapid succession; the subject’s task is to indicate their temporal order – i.e., identify a *before-after* relation. The correctness of such judgments reflects temporal acuity, necessary for the identification of incoming events in analytical, sequential information processing (von Steinbüchel et al., 1999; Szymaszek et al., 2009; Szelag et al., 2011; Babkoff and Fostick, 2013, 2017; Fostick and Babkoff, 2013a). Accordingly, it has been postulated that patterning in a time window of some tens of milliseconds is controlled by a neural mechanism characterized by time limits of approximately 30 ms (Pöppel, 1994, 1997). This temporal ordering ability directly indicates the distinct nature of TIP.

Auditory Temporal-Order Threshold (ATOT) can be used as an index of temporal acuity (i.e., the efficiency of identifying event ordering) and can be measured using a TOJ paradigm. ATOT is defined as the shortest time gap (in milliseconds) between two sounds presented in rapid succession with an Inter-Stimulus Interval (ISI) of some tens of milliseconds necessary to identify their *before-after* temporal relation with at least 75% correctness (Szelag et al., 2011, 2014, 2015b; Bao et al., 2013, 2014). An auditory TOJ paradigm may employ various measurement procedures. Subjects may be presented with a sequence of tones of different frequencies delivered monaurally or binaurally (Ben-Artzi et al., 2005, 2011; Bao et al., 2014), two or four stimuli sequences of clicks, tones or syllables (Ulbrich et al., 2009), as well as the same auditory stimuli (e.g., tone bursts or clicks) presented monaurally with a difference in the time of arrival of the stimulus at the left and right ear (Fink et al., 2005, 2006; Szymaszek et al., 2009; Szelag et al., 2011; Bao et al., 2013, 2014). Accordingly, the spatial TOJ task reflects a situation where two identical stimuli are presented monaurally in an alternating presentation mode and the task is to identify the ear to which the first stimulus was delivered and the ear to which the second was delivered (*left-right* or *right-left*). In contrast, in the spectral TOJ task, two different stimuli (e.g., high and low tones) are presented binaurally and the task is to indicate the order of their occurrence (*high-low* or *low-high*). It should be stressed that, in addition to temporal processing, these two TOJ tasks also involve task-specific perceptual processes, which are the topic of the present study.

Starting from the seminal papers by Hirsh and Sherrick, 1961) and Efron (1963), temporal resolution ability has been widely applied in experimental studies to assess millisecond timing efficiency in both normal subjects and various clinical subgroups. Hence, reliable measurement procedures are very important for drawing reasonable conclusions about a subject’s information processing.

The basic and still open question is: how do our brains process temporal information in this time domain? According to the hypothesis proposed by Pöppel (1997, 2009), visual or acoustic stimuli processed within a time window of less than ca. 30 ms are treated as co-temporal or a-temporal. Thus, their *before-after* relation cannot be established. For healthy young subjects to perceive the temporal order of two distinct events correctly, the minimum delay between these stimuli must exceed ca. 30 ms.

Several authors claim that one central mechanism which samples time discretely is responsible for the assessment of temporal order both within and across sensory systems. Evidence supporting such a hypothesis comes from experimental studies indicating similar threshold values both across sensory systems and within sensory modalities, including the auditory system. The main evidence for a central mechanism was provided by Hirsh and Sherrick, 1961). They found the same threshold of 17 ms for temporal ordering in different sensory systems, as well as for cross-modal comparisons. Other authors have also found evidence for this central mechanism hypothesis, e.g., Mills and Rollman (1980), Pöppel (1994, 1997), Wittmann (1999), Szelag et al. (2004a), Babkoff et al. (2005), Ben-Artzi et al. (2005), Fink et al. (2005, 2006), Bao et al. (2014). These studies, which employed various types of sensory stimuli and procedures in healthy young controls, indicated thresholds for temporal ordering between 20 and 60 ms. Furthermore, some of these authors reported unique response patterns produced by different TOJ paradigms (Fink et al., 2005, 2006; Szymaszek et al., 2006, 2009; Szelag et al., 2011; Bao et al., 2013, 2014; Fostick and Babkoff, 2013b).

In more recent papers using different variants of experimental TOJ tasks and various subject subpopulations, evidence has suggested that TOJ on the millisecond level may be influenced by various procedures and subject-related factors, the most important of which seem to be the type of stimuli used, presentation mode, age, cognitive status, gender, as well as neurodevelopmental or neurodegenerative disorders (for the overview see von Steinbüchel et al., 1999; Wittmann and Szelag, 2003; Szelag et al., 2004b, 2010, 2011, 2015a,b; Szymaszek et al., 2009, 2018; Teixeira et al., 2013; Matthews and Meck, 2014; Oron et al., 2015). Existing studies have also confirmed individual differences in TIP at this processing level in healthy volunteers of various ages (Szymaszek et al., 2009; Szelag et al., 2011; Bao et al., 2013, 2014).

Of course, in a given experimental situation, subject-related factors co-exist with procedure-related influences. But the relations between these complex factors, critical for the measurement of resolution ability in an individual, are still an open question. Furthermore, their neural basis is still a poorly understood area of psychology and neuroscience. One of the problems in these studies is clarifying the degree to which the applied paradigms are sensitive to pure temporal processes and to stimulus-related, procedural, and other influences. As previous studies raise questions about the relationships between different paradigms, in this study we concentrate on the relationship between auditory spatial and spectral TOJ paradigms tested in the same subject pool with comparable procedures, considering also the test–retest repetition of measurements in consecutive sessions.

There are considerable data available in the literature indicating age-related decline of temporal resolution ability in processing in the millisecond domain (e.g., Fitzgibbons and Gordon-Salant, 1998; Fink et al., 2005; Kołodziejczyk and Szelag, 2008; Ulbrich et al., 2009; Fostick and Babkoff, 2013a). This has been interpreted as part of the general deterioration of mental functions in advancing age, even in normal healthy elderly individuals who do not suffer from any neurodegenerative problems (e.g., Szelag et al., 2010; Nowak et al., 2016). One challenge for recent TIP studies has been to learn how the procedures used influence temporal acuity in different TOJ tasks. This topic has been mostly explored in young volunteers. For example, the recent meta-analysis by Fostick and Babkoff (2017) focused on a comparison of ATOT values obtained using the auditory spectral vs. spatial TOJ tasks. This comparison was based on the threshold distribution characteristics of 388 subjects tested in 13 spectral TOJ experiments and of 222 subjects tested in 9 spatial TOJ experiments. However, the pool of subjects in all meta-analyzed experiments comprised only young individuals (university students) aged from 20 to 34 years (Fostick and Babkoff, 2017; see characteristics of the meta-analyzed participants provided in Tables 1, 4 of this report). Despite many existing studies on age-related decline in TOJ, no definitive explanation of procedure-related influences on ATOT values has been evidenced in elderly (Fink et al., 2005; Szymaszek et al., 2006, 2009; Ulbrich et al., 2009).

On the other hand, our previous study on auditory TOJ using both spatial and spectral tasks in listeners aged from 20 to 69 years concentrated mostly on differences between mean ATOT values between particular age groups (Szymaszek et al., 2009), whereas direct between-tasks comparisons for ATOT distributions within particular age groups were not analyzed. A similar approach was explored by Fink et al. (2005). Furthermore, another paper by Fink et al. (2006) reported lower ATOTs in the spectral task than in the spatial task, but the subject pool comprised individuals aged between 21 and 50 years of age analyzed in a single group.

### Aim of the Study

To learn more about procedure-related influences on temporal acuity in advancing age, in the present study we test the effect of spatial vs. spectral paradigms on the auditory perception of temporal ordering in a relatively large group of elderly listeners. We aimed to extend existing findings about procedure-related effects on temporal acuity in elderly listeners and to clarify whether the expected influences are similar to those indicated in previous literature studies (Fink et al., 2005, 2006; Szymaszek et al., 2009). We compare directly, in the same sample of subjects, the response distributions obtained using spatial vs. spectral TOJ paradigms.

The identification of such procedure-related differences may increase our understanding of TIP in elderly. We therefore ask three following questions: (1) Do the obtained ATOTs differ between spectral and spatial TOJ tasks? (2) What are the distributions of the subjects’ data on these two tasks? (3) Do results on these two tasks have high test–retest reliability? Similarities between performances on these two TOJ tasks would verify the hypothesis of the existence of a common timing mechanism which in elderly operates independently of the task (spatial or spectral).

## Materials and Methods

This study was approved by the local Ethical Commission at the University of Social Sciences and Humanities (permission no 1/2017, registered as 2 /I/ 16-17) and was in line with the Declaration of Helsinki. All participants provided their written informed consent prior to the study.

### Participants

We tested 40 elderly subjects (36 females and 4 males) aged from 62 to 78 years (*M* = 67.4, *SD* = 3.6). They were recruited from the Warsaw area by advertisements in newspapers, on the internet, as well as at Universities of the Third Age (U3A) and in various local community centers. All subjects were right-handed native Polish speakers. They reported no history of neurological or psychiatric disorders, head injuries in the past, systemic diseases, or the use of medications affecting the central nervous system. The above-mentioned inclusion criteria were verified in a brief interview with each subject.

All participants were screened for normal hearing levels (American National Standard Institute, 2004) using pure-tone audiometry (Audiometer MA33, MAICO) at the following frequencies: 250, 500, 750, 1000, 1500, 2000, and 3000 Hz, which covers the frequency spectrum used in the presented stimuli. To screen for dementia or depression, all participants completed the Mini-Mental State Examination (MMSE; Folstein et al., 2001) and the Geriatric Depression Scale (GDS; Sheikh and Yesavage, 1986) prior to the TOJ task. Inclusion criteria were: a score of at least 27 points on the MMSE (*M* = 28.8, *SD* = 1.1) and a score of 5 or fewer points on the GDS (*M* = 2.5, *SD* = 1.5). All subjects reported having between 11 and 18 years of education.

These inclusion criteria allowed us to expect that the participants were in relatively good physical and mental health. It may be assumed, therefore, that they exhibited the level of cognitive functioning typical of normal healthy aging.

### Stimuli and Presentation Modes

As noted above, two TOJ tasks were used which differed in both type of stimuli and stimulus presentation modes (Szymaszek et al., 2009; Bao et al., 2014; Nowak et al., 2016). Both tasks used paired acoustic stimuli presented in rapid succession. The stimuli were generated by a computer with a Realtek ALC3246 sound controller using Waves MaxxAudio Pro software on Philips SHP8500 headphones at a comfortable listening level. Two stimuli within each pair were separated by various ISIs reflecting the time gap between the *offset* of the first stimulus and the *onset* of the second stimulus. The duration of the ISIs varied during the experiment according to a pre-defined adaptive algorithm (see below for a more detailed description).

#### In the Spatial Task

The presented pairs consisted of two rectangular pulses (clicks) of 1 ms duration each, which were presented monaurally in an alternating stimulation mode, i.e., one click was presented to one ear followed by another click to the other ear. The subject’s task was to verbally report the temporal order of the two successive stimuli within each pair. Two alternative responses were possible: *left-right* or *right-left.*

#### In the Spectral Task

**FIGURE 1:**
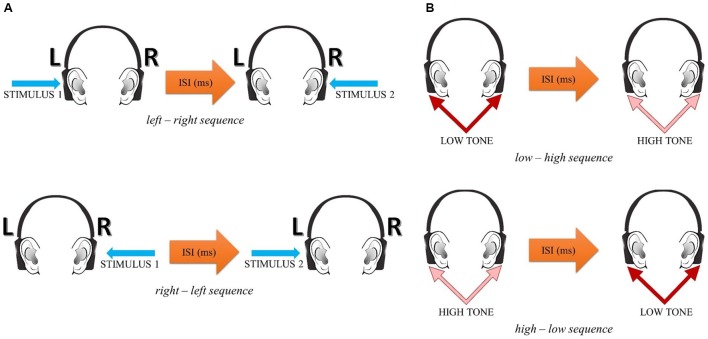
The experimental setup in the spatial **(A)** and spectral **(B)** tasks.

The presented pairs consisted of two 10 ms sinusoidal tones – a low tone of 400 Hz and a high tone of 3000 Hz. The rise-and-fall time of each tone was 1 ms. The two tones within each pair were adjusted to equal loudness on the basis of isophones. The binaural stimulus presentation mode was used, i.e., each tone pair was presented to both ears with various ISIs between the two tones in each pair (similar to the spatial task, see above). The subjects were asked to verbally report the temporal order of the two successive tones within each pair. Two alternative responses were possible: *low-high* or *high-low.* The experimental situation is displayed in Figure 1.

### Procedure

The experiment was conducted in a soundproof room at the Laboratory of Neuropsychology at the Nencki Institute.

To focus the participant’s attention on the upcoming task, each pair of stimuli was preceded by a warning signal delivered binaurally 1 s before the first stimulus within each pair. Then, the paired stimuli were presented monaurally (in the spatial task) or binaurally (spectral task). After each presentation, subjects reported the order of the two stimuli in the presented pair.

Prior to the collection of data, each participant was given a verbal instruction by the experimenter and, then, presented with a few practice trials consisting of pairs with a relatively long ISI. In these practice trials, feedback was given on the subject’s correctness after each answer. All participants performed these practice trials satisfactorily. Next, the proper measurement started and no feedback on correctness was given.

We used an adaptive algorithm based on maximum likelihood estimation to measure the subjects’ ATOTs in both tasks. The implementation of the algorithm for testing elderly listeners studied here was based on the literature reports by Treutwein (1997), Fink et al. (2005, 2006) and Wittmann and Szelag (2003), as well as on our previous studies (Szymaszek et al., 2009; Szelag et al., 2011; Bao et al., 2013, 2014; Nowak et al., 2016). The algorithm consisted of two parts. In the first part, the participant responded to 20 trials comprising paired stimuli presented with 10 fixed ISIs of varying durations. They were presented first in decreasing and, subsequently, in increasing order (i.e., up and down) according to pre-defined rules. The ISIs in the spatial task ranged from 160 ms to 1 ms (changing in 18 ms steps), and in the spectral task from 240 ms to 1 ms (changing in steps of 27 ms). These different testing ranges in the spatial and spectral tasks resulted from our previous observations, indicating different order thresholds in these two tasks in elderly subjects.

After completion of these 20 trials, based on the correctness of the subject’s responses, the program calculated the ISI value for the initial trial in the second part of testing at the 75% probability of correct responses according to maximum likelihood estimation (Treutwein, 1997). In the second part of testing, 50 trials were presented. In each of these 50 trials, the ISI was adjusted adaptively: it decreased after each correct response and increased after each incorrect response. The exact values of decreased or increased ISIs were randomly selected from a pre-defined range which varied depending on the ISI being tested. To ensure accurate and precise assessment, decremental steps were 0.5–5% of the ISI value of the previous trial, while increments were 10–20% of the previous ISI value. On the basis of 70 completed trials (i.e., 20 trials in the first part of testing and 50 trials in the second part), the ATOT value for each participant was taken as the mean of the estimated likelihood, calculated at 75% probability level of correct responses (Treutwein, 1997).

The measurement was conducted with each subject individually in two separate sessions (Session 1 and Session 2), separated by a break of a few days. In each session, both the spatial and spectral tasks were completed. The order of tasks within each session was constant: first the spatial task was conducted followed by the spectral task. The TOJ measurement lasted approximately 10 min for each task. Each session lasted approximately half an hour.

## Results

Thresholds were estimated for all participants for both TOJ tasks based on performance in Session 1 and Session 2. As the temporal information was processed from the onset of the first stimulus within a pair and different stimulus durations were used in the spatial (1 ms) and spectral (10 ms) tasks, the ISI values were replaced by Stimulus-Onset Asynchrony (SOA) values to compare the performance between these two tasks. Such procedure was applied in many previous reports (Fink et al., 2005, 2006; Ulbrich et al., 2009, see Table 1 in this report), including our studies (Szymaszek et al., 2009). SOA reflects the time between the *onset* of the first stimulus and the *onset* of the second stimulus within a pair and gives the ATOT values analyzed for each task and session (see Introduction for the definition of ATOT). For example, a stimulus duration of 1 ms clicks (monaural task) and an ISI of 60 ms gives a SOA of 61 ms. But the same ISI value of 60 ms using paired tones of 10 ms duration (binaural task) results in a SOA of 70 ms. Therefore, the analyzed SOA values were found by adding the stimulus duration (either 1 ms for the spatial task or 10 ms for the spectral task) to the ISI at which there was a 75% probability of correct responses.

### Distribution of ATOTs in Spatial and Spectral Tasks

**FIGURE 2:**
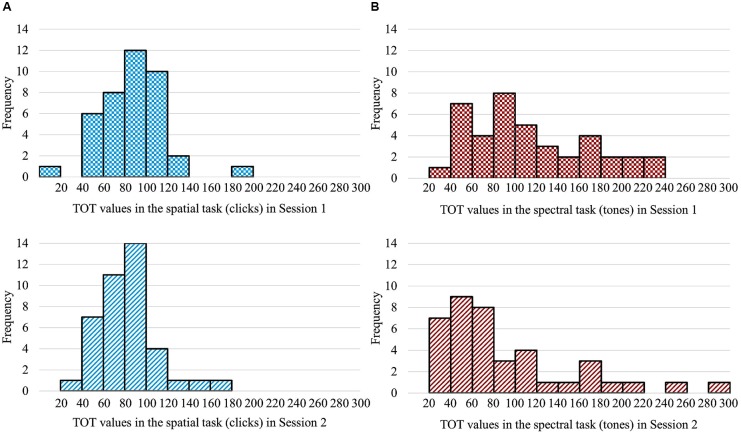
Different distributions of ATOT data in particular subjects across the two TOJ tasks in consecutive sessions. *Additional explanations:* **(A)** The distribution of ATOT data in the spatial task was Gaussian (*skewness: 0.70 and 0.65; kurtosis: 2.85 and 0.95; Shapiro–Wilk test: 0.95 and 0.96*). **(B)** The distribution deviated significantly from the Gaussian in the spectral task (*skewness: 0.60 and 1.52; kurtosis: –0.58 and 2.02; Shapiro–Wilk test: 0.94, *p* < 0.05 and 0.83, *p* < 0.001*).

Examining the data obtained from particular subjects, we observed important differences in the distribution of ATOT values for the spatial and spectral tasks (Figure 2). In the former case (Figure 2A), the data indicated no significant deviation from the Gaussian distribution across subjects and sessions. In contrast, in the spectral task (Figure 2B), the distribution of ATOTs deviated significantly from the Gaussian and was spread out more to the right (based on visual inspection, values of skewness and kurtosis, as well as results of the Shapiro–Wilk test; see Figure 2 legend for more details). Such a dissociation in the data distributions of the two tasks was observed in both sessions.

### Comparison of ATOTs in Spatial and Spectral TOJ Tasks

Descriptive statistics of the ATOT values obtained in the spatial and spectral TOJ tasks are presented in Table 1.

**Table 1:** Descriptive statistics of the ATOT values (in ms) in two consecutive sessions for spatial (monaural presentation of paired clicks) and spectral (binaural presentation of paired tones) tasks.

	Spatial task			Spectral task		
	Session 1	Session 2	Mean of two sessions	Session 1	Session 2	Mean of two sessions
**Median**	90	85	83	102	66	92
**Range**	19–191	29–162	32–177	38–231	23–298	35–264
**Mean**	88	83	85	114	91	103
**SEM**	5	4	4	9	10	8
**SD**	30	27	26	55	64	50

This table shows that the ATOTs were, in general, lower in Session 2 than in Session 1 (reflecting better performance), independent of the task. However, the between-session differences were more pronounced in the spectral task than in the spatial task.

Because of the between-tasks differences in the data distribution (explained above), to directly compare the performance on these two tasks using parametric statistical analysis, we transformed the ATOT data by square root extraction, resulting in the distribution of ATOT data approaching Gaussian. Such a transformation is recommended in the literature for the spread more to the right distributions.

Further statistical analysis was performed, therefore, using a 2-way ANOVA with repeated measures including ‘Task’ (spatial vs. spectral) and ‘Session’ (1 vs. 2) as within-subjects variables. Significance values were assumed at *p* < 0.05 corrected by the Bonferroni test applied to the observed main effects and interactions. The effect sizes, indexed by partial-eta squared statistics (η^2^), are reported for all significant effects.

**FIGURE 3:**
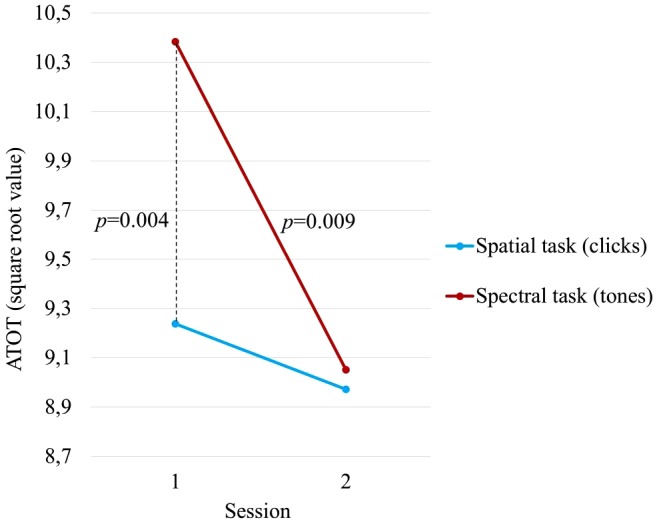
The main effect of ‘Session’ modified by the interaction ‘Session x Task’ indicated in a 2-way ANOVA performed on transformed square root values of ATOT.

The ANOVA revealed a main effect of ‘Session’ [*F*(1,39) = 8.156, *p* = 0.007, η^2^ = 0.173] modified by the interaction ‘Session x Task’ [*F*(1,39) = 4.371, *p* = 0.043, η^2^ = 0.101]. The main effect of ‘Task’ was non-significant. These relationships are presented in Figure 3. This interaction resulted from the different effect of ‘Session’ in the two tasks. In the spatial (clicks) task, ATOTs were relatively stable across the two consecutive sessions and the difference between sessions was non-significant. In contrast, in the spectral (tones) task, the ATOTs in Session 2 were significantly (*p* = 0.009) lower than in Session 1, indicating improved performance. Furthermore, significant differences between the tasks were observed only in Session 1 (*p* = 0.004), being non-significant in Session 2.

### Reliability of TOJ Measurement in Two Consecutive Sessions

In addition, to verify the test–retest reliability of both spectral and spatial tasks, Pearson correlation analysis was performed. The correlation coefficients (controlling for the subjects’ age) of the transformed ATOTs (see above) in Session 1 and Session 2 reached statistical significance in both tasks, indicating a reliable measurement of ATOT on the two TOJ tasks used in the present study. However, the between-session coefficient in the spatial task had higher value than that in the spectral task (*r* = 0.61, *p* < 0.001 vs. *r* = 0.40, *p* = 0.011, respectively). Furthermore, within-session correlation coefficients between two tasks reached statistical significance only in Session 1 (*r* = 0.43, *p* = 0.006), being non-significant in Session 2 (*r* = 0.30, *p* = 0.062).

## Discussion

### Comparison of Spatial and Spectral TOJ Tasks

The main finding of the present study was the identification of differences in the elderly in the performance on two TOJ tasks (spatial and spectral) which utilize temporal resolution. Differences were observed in both the threshold distribution on these two tasks and the ATOT values obtained in two sessions. Based on the threshold distributions, we observed a dissociation in the performance on these two TOJ tasks in subjects aged between 62 and 78 years. In the elderly studied in this experiment, results on the spatial TOJ task had a Gaussian threshold distribution (Figure 2A) accompanied by a relatively stable performance across two sessions (Table 1 and Figure 3). In contrast, the spectral task was characterized by a non-Gaussian distribution (Figure 2B) and a significant lowering of ATOT values in Session 2 – indicating improved performance (i.e., a shorter gap being necessary to correctly order incoming stimuli; Table 1 and Figure 3).

At this point one should refer to data meta-analyzed by Fostick and Babkoff (2017) indicating also the task-related dissociation in the distribution of ATOT data. Specifically, a Gaussian threshold distribution was reported in the spatial task, while the spectral TOJ thresholds distribution was skewed to the right. Furthermore, a similar mean of ATOTs in these two tasks (78.21 ms vs. 78.34 ms, respectively) was reported but the range of mean ATOTs in the spectral task (31.95–116.13 ms) was broader than in the spatial task (56.84–93.23 ms), reflecting the higher variability in the former case. Referring to this literature study, a similar pattern of task-related dissociation in the threshold distribution was evidenced in both elderly (studied here) and young listeners (investigated in the previous reports). It shows that, despite the substantial age-related decline in temporal acuity evidenced in previous literature studies (see papers cited in the Introduction), the effect of task specificity on the threshold distribution remains relatively stable across one’s entire lifespan. The question is which processes may be responsible for such task-related differences in elderly?

The perceived order of two stimuli presented in rapid succession may reflect not only the temporal template, but also task-specific processes as well as different stimulus- and procedure-related influences. Therefore, the above dissociation can be also explained in terms of differences in the very structure of these two tasks. Our data indicated that experimental factors which constrain the subject’s responses affect the measured ATOT values additively. These two tasks seem to be performed using different perceptual strategies implemented in the auditory processing within the nervous system. The TOJ paradigm, rooted in auditory perception, may not just be associated with a timing mechanism free of any procedure-related (non-temporal) influences. Despite the rapid presentation of stimuli with short ISIs in both tasks, there were important differences between these tasks. In the spatial task, the two clicks were identical in all of their characteristics covering an identical frequency spectrum delivered asynchronously to each ear. In contrast, the spectral task employed two tones differing in frequency (400 vs. 3000 Hz) which were delivered asynchronously to both ears. Each of these two tasks involved task-specific strategies in addition to the temporal processes.

Referring to the previous reports on auditory perception, two tones of different pitches may be perceived as a single tone sound of rising or falling frequency (Micheyl et al., 2007; Deike et al., 2010; Selezneva et al., 2018). This hypothetical phenomenon of auditory streaming or frequency modulation reflects the specific sensory integration process within the auditory system, which is likely also involved in auditory TOJ. This may reflect a specific perceptual bias toward integrated auditory perception of tones presented in rapid succession. Accordingly, sequences of two tones might be perceived as a single frequency-modulated tone glide either *rising* (*low-to-high*) or *falling* (*high-to-low*), removing the need to identify the first and second tone. In the literature, the phenomenon of auditory streaming has been studied using sequences of tones in both humans and animals, and several theories about its neural basis have been proposed (Hartmann and Johnson, 1991; Rauschecker, 1998; Micheyl et al., 2007; Snyder and Alain, 2007; Selezneva et al., 2018).

Because of these processes, the two tones within a sequence could be integrated into a single percept at short SOA, so their *before-after* relation could be identified based on a frequency-modulated pattern (*rising* or *falling*) rather than on the detection of the temporal order of separate stimuli (a *before-after* relation). As a consequence, some individuals might reproduce the order from such modulated tone glides and thus circumvent the need to identify the first and second stimulus within a presented pair. As there are probably individual differences in ability to perceive auditory streaming, the results distribution of the spectral task had higher variability than that of the spatial task (see Figure 2). Judgment is based on the above strategy to a greater extent in the spectral task, which seems to depend more than the spatial task on auditory streaming and frequency modulation (which have greater effectiveness at shorter SOA). The spectral paradigm, therefore, seems to be more constrained by the perceptual strategies associated with tone processing. In contrast, the streaming strategy cannot be used in spatial auditory TOJ because the spatial task employs clicks of identical pitch, rather than tones differing in pitch.

To summarize, different perceptual strategies seem to be engaged in TIP when clicks or tones are used to order sounds which are presented asynchronously in rapid succession. The question remains about the relation between the threshold values in the spatial and spectral tasks. In our study, we found significant differences between tasks only in Session 1 resulting from the better temporal acuity in the spatial than the spectral task (Figure 3). This pattern of relationships is not in line with previous reports. In our previous study (Szymaszek et al., 2009), the spectral task resulted in shorter mean ATOT values (76 ms) than did the spatial task (88 ms) in a group of 16 listeners aged from 60 to 69 years. A similar relationship was reported by Fink et al. (2006, see Table 1 in this report) who found thresholds of 57.54 ms vs. 31.24 ms in the spatial and spectral tasks, respectively, in a sample of 50 participants (aged between 21 and 50 years). Moreover, both the above literature studies did not report session-related differences between tasks.

We think that this reversed relation between ATOTs could result from the shape of the data distributions obtained on these two tasks. In recent literature, some researchers indicated that a relatively large number of participants performed a spectral TOJ task (separating successively presented tones) with very short SOA (Fink et al., 2006, Figure 1; Fostick and Babkoff, 2013b, 2017, Figure 1). Thus, the higher skewness of the spectral threshold distribution was mainly due to a large number of participants with ATOTs shorter than 20 ms. For example, Fink et al. (2006) indicated that more than 20 out of 50 participants (aged 21–50 years) had thresholds of 10–20 ms. In our study, as shown in Figure 2B, the number of participants with short SOAs was smaller. It is likely that many participants had higher ATOTs in the spectral task in this experiment due to their being older (aged from 62 to 78 years) than the participants in the above literature reports.

The question is whether the perceptual constraints of timing mechanisms reported here are age-specific, thus, characteristic for elderly or age-independent, thus, evidenced across the broader lifespan. It may be concluded that there is some significant decline in auditory streaming ability and frequency modulation in late adulthood, despite all participants having had normal hearing levels. To our knowledge, no apparent role of age in auditory streaming has been previously reported. Further studies are needed to explore these implications about the relationships between effects of temporal and perceptual (non-temporal) processes on measured threshold values in particular age groups.

### Comparison of the Two Consecutive Sessions

Lower ATOT values were usually observed in Session 2, rather than in Session 1, which was reflected in the significant main effect of ‘Session’ (*p* = 0.007, see ANOVA results). This may have been due to training or adaptation to the task after two consecutive repetitions of the measurement separated by a break of a few days. This learning effect may correspond not only to improved TIP (i.e., temporal acuity of information processing), but also to changes in concomitant non-temporal processes involved in the TOJ task, discussed above. The possibility of improved TIP was also reported in previous studies and is applied in new cognitive therapy methods based on the transfer of improvement from the trained time domain to the cognitive domain, which was not trained during the intervention (e.g., Tallal et al., 1996; Szelag et al., 2015a; Szymaszek et al., 2018).

The most important result of our study was a strong dissociation in the magnitude of this learning effect between the two tasks, reflected in the ‘Session x Task’ interaction (*p* = 0.043). Whereas a huge improvement in Session 2 (*p* = 0.004, Bonferroni test) was found in the spectral task, in the spatial task this difference was non-significant (Figure 3). To explain this dissimilar effect of session, we refer once again to the use of specific perceptual strategies based on frequency modulation and auditory streaming in the auditory processing of tones discussed above. We hypothesize that repeated measurement may foster better application of the auditory streaming strategy in the spectral task, which would not have occurred in Session 1 likely because of the task novelty.

The use of the aforementioned *rising* vs. *falling* two tone glides, rather than the identification of consecutive tones, may result in significantly lowered ATOTs in Session 2 as compared to Session 1. As in the elderly participants studied here, this perceptual strategy in the spatial task (identical clicks presented monaurally) seems less helpful, the learning effect was rather small (median ATOT of 90 vs. 85 ms in Sessions 1 and 2, respectively, see Table 1) and statistically non-significant. It probably reflects more the improved TIP. To summarize, we are of the opinion that the test–retest comparisons in the two TOJ tasks indicate both improved TIP and the use of an auditory streaming perceptual strategy in the elderly participants.

### Reliability of ATOT Measurements and Practical Implications for Future Studies

Pearson correlations between the ATOTs obtained in the two consecutive sessions in each task (controlling for the subjects’ age) indicated moderate significant correlation coefficients. Such positive correlations between the two sessions seem important for understanding how our brains create the inner experience of time and whether we are equipped with a central millisecond timing mechanisms (see below). This would suggest that both paradigms studied here constitute reliable measurement tools for the replicable assessment of sequencing abilities measured by auditory TOJ.

The correlation coefficients had rather moderate values in both tasks (i.e., *r* = 0.61, *p* < 0.001 vs. *r* = 0.40, *p* = 0.011 in the spatial and spectral tasks, respectively), which may suggest the influence of intra-individual variability on the measured indices of temporal resolution in the two consecutive sessions. Such intra-individual variability might be a result of the contribution of other cognitive processes (e.g., perception, attention, working memory, decision making, etc.) to TOJ. Specifically, the lower correlation coefficient accompanied by a lower significance level on the spectral task than on the spatial task may be due to the involvement of perceptual strategies associated with the auditory perception of tonal stimuli discussed above. The effect of these strategies seems to be more pronounced in the spectral task because of the specific processes (auditory streaming) involved in tone perception. We are of the opinion that such extra effects, which co-exist with TIP, may generate additional variability in addition to the intra-individual variability of TIP. Hence, there was a lower correlation coefficient and a lower level of significance on the spectral task than on the spatial task.

Another support for the involvement of extra non-temporal processes in TIP comes from correlation coefficients within a time point between tasks which reached statistical significance only in Session 1 being non-significant in Session 2. It may result from the involvement of more pure timing processes in Session 1 which in Session 2 are constrained by additional non-temporal perceptual processes related to auditory streaming in the spectral task in Session 2 (see Figure 3).

The discussion about the reliability of ATOT measurement using these two paradigms may be important for future clinical applications of TOJ in comparisons between normal samples and various clinical subpopulations. As mentioned in the Introduction, many patient groups show deficient timing accompanying a decline in cognitive processes. Thus, accurate and reliable measurement tools for the assessment of TIP (including temporal resolution in the millisecond domain) are necessary to provide measurements for diagnostic purposes. The obtained indices should be considered with caution because differences between groups may not necessarily reflect deficient temporal acuity, but rather difficulties using the same perceptual strategies as the controls.

On the basis of comparisons between the two sessions reported here, we might suggest some practical implications for future studies to increase their testing validity. On the spatial task, we postulate that the evaluation of TIP efficiency should not be based on a measurement from a single testing session because some learning or adaptation effects (although non-significant) were visible between Session 1 and 2 (see Figure 3). To reduce inaccurate assessments, the measurement should be repeated a few times in consecutive sessions so that mean ATOTs reflect more veridical temporal acuity. In contrast, in the spectral task the absolute ATOTs having been significantly higher in Session 1 than in Session 2, as well as the strong learning effect observed in Session 2 (Figure 3), shows that, in future applications, TOJ indices in particular sessions should be considered rather as a separate normative data characteristic for a given test repetition.

The procedure-related differences reported here give rise to another problem for which TOJ tasks could be suitable for use in future studies. Given that the spatial TOJ task probably better reflects TIP without the additional influences of perceptual strategies, this task might be recommended for a more veridical assessment of TIP efficiency in individuals. Additional influences generated by non-temporal perceptual strategies associated with auditory perception of presented stimuli might blur the genuine timing properties.

### One Central Mechanism vs. Various Task-Dependent Mechanisms

The final problem to be considered here concerns the conceptualization of processing mechanisms controlling the perception of succession and temporal resolution. As described in the Introduction (above), two distinct hypotheses on this issue exist in the literature. One of them assumes a central timing mechanism responsible for TOJ, independent of the paradigm used (Hirsh and Sherrick, 1961); Efron, 1963; Mills and Rollman, 1980; Pöppel, 1994, 1997, 2009; Wittmann, 1999; Szelag et al., 2004b; Babkoff et al., 2005; Ben-Artzi et al., 2005; Bao et al., 2014). The other hypothesis suggests a paradigm-specific and strongly procedure-dependent mechanism controlling this ordering ability (Fink et al., 2005, 2006; Szymaszek et al., 2006, 2009; Szelag et al., 2011; Fostick and Babkoff, 2017).

To better understand the neural basis underlying temporal ordering, one should refer to the hypothesis about time windows for temporal integration mentioned in the Introduction (Pöppel, 1994, 1997). Some evidence supports the notion of temporally discrete information processing within a time window of some tens of milliseconds. This assumption leads to the discussion of the theoretical model of temporal ordering. The neural mechanisms responsible for temporal resolution seem to be based in neuronal oscillatory activity, as evidenced in electrophysiological studies which indicate a periodicity of about 40 Hz (between 30 and 80 Hz; VanRullen and Koch, 2003; Oron et al., 2015; see also Benasich et al., 2008 for a review). Thus, one oscillation period has ca. 25 ms duration. According to Pöppel (1997, 2009), a *before-after* relation can only be perceived if the two stimuli occur within at least two successive oscillatory periods. Thus, to identify the *before-after* relation of stimuli presented in rapid succession, they must be separated by a time gap of some tens of milliseconds. There is strong evidence that spontaneous (or stimulus triggered) gamma band oscillations, presumably corresponding to ATOT, play an important role in human cognition (VanRullen and Koch, 2003).

On the basis of our results, which indicate a clear dissociation in performance between the spatial and spectral TOJs, one might conclude that these data do not support the hypothesis on the central mechanism which controls the ability to sequence stimuli, as evaluated with various stimuli and procedures. This conclusion, however, should be drawn with caution. Referring to the neuro-oscillatory activity which probably constitutes the physiological basis for TIP in the millisecond range (see above), one may assume that the time limits of the underlying mechanism can be modified by different non-temporal processes, including perceptual strategies in the spatial and spectral TOJs used in this study. In the former case, performance seems to reflect more the efficiency of the genuine timing, whereas in the latter case, it may correspond to auditory streaming and frequency modulation occurring within the auditory pathway. As distinct processes for detecting the temporal order are evoked by each task, different absolute threshold values should be expected, meaning that we cannot rule-out the hypothesis of a single central mechanism forming the basis of temporal resolution measured with behavioral methods. The ATOTs obtained in the spectral task, therefore, seem to reflect an interaction between the genuine timing and cognitive processes related to perception of auditory stimuli. The idea of temporal processes constrained by perceptual non-temporal task specific processes can be supported in our study by both the mean ATOT values and correlation coefficients (between- and within-session).

Finally, we are of the opinion that temporal acuity and sequencing abilities are based in neuronal oscillatory activity. However, the absolute thresholds measured in auditory TOJ tasks are stimulus-dependent, procedure-related, and influenced by the perceptual strategies used by participants.

## Author Contributions

ES conceived and designed the study, analyzed and interpreted the data, wrote the manuscript, and was responsible for the final version of the manuscript. KJ and MP recruited the subjects and acquired, analyzed, and interpreted the data. AS and HB interpreted the data and wrote the manuscript.

## Conflict of Interest Statement

The authors declare that the research was conducted in the absence of any commercial or financial relationships that could be construed as a potential conflict of interest.
